# Regulatory Elements Located in the Upstream Region of the *Rhizobium leguminosarum rosR* Global Regulator Are Essential for Its Transcription and mRNA Stability

**DOI:** 10.3390/genes8120388

**Published:** 2017-12-15

**Authors:** Kamila Rachwał, Paulina Lipa, Iwona Wojda, José-María Vinardell, Monika Janczarek

**Affiliations:** 1 Department of Genetics and Microbiology, Institute of Microbiology and Biotechnology, Faculty of Biology and Biotechnology, Maria Curie-Skłodowska University, Akademicka 19 St., 20-033 Lublin, Poland; rachwal.kamila@gmail.com (K.R.); paulina.lipa56@gmail.com (P.L.); 2 Department of Immunobiology, Institute of Biology and Biochemistry, Faculty of Biology and Biotechnology, Maria Curie-Skłodowska University, Akademicka 19 St., 20-033 Lublin, Poland; wojda@hektor.umcs.lublin.pl; 3 Department of Microbiology, Faculty of Biology, University of Sevilla, Avda. Reina Mercedes 6, 41012 Sevilla, Spain; jvinar@us.es

**Keywords:** *rosR*, gene expression, transcription regulation, RNA secondary structures, RNA stability, *Rhizobium leguminosarum*, symbiotic bacteria

## Abstract

*Rhizobium leguminosarum* bv. *trifolii* is a soil bacterium capable of establishing a symbiotic relationship with clover (*Trifolium* spp.). Previously, the *rosR* gene, encoding a global regulatory protein involved in motility, synthesis of cell-surface components, and other cellular processes was identified and characterized in this bacterium. This gene possesses a long upstream region that contains several regulatory motifs, including inverted repeats (IRs) of different lengths. So far, the role of these motifs in the regulation of *rosR* transcription has not been elucidated in detail. In this study, we performed a functional analysis of these motifs using a set of transcriptional *rosR-lacZ* fusions that contain mutations in these regions. The levels of *rosR* transcription for different mutant variants were evaluated in *R. leguminosarum* using both quantitative real-time PCR and β-galactosidase activity assays. Moreover, the stability of wild type *rosR* transcripts and those with mutations in the regulatory motifs was determined using an RNA decay assay and plasmids with mutations in different IRs located in the 5′-untranslated region of the gene. The results show that transcription of *rosR* undergoes complex regulation, in which several regulatory elements located in the upstream region and some regulatory proteins are engaged. These include an upstream regulatory element, an extension of the -10 element containing three nucleotides TGn (TGn-extended -10 element), several IRs, and PraR repressor related to quorum sensing.

## 1. Introduction

*Rhizobium leguminosarum* bv. *trifolii* is a soil bacterium that establishes nitrogen-fixing symbiosis with clover (*Trifolium* spp.). However, in the absence of compatible host plants, this micro-organism must often exist for a long period of time in the soil, where it is exposed to various environmental conditions. To adapt to these conditions, rhizobia have developed a wide range of strategies which allow them to survive in the soil. Among these adaptations, composition of the bacterial envelope, synthesis of surface polysaccharides, cell motility, and quorum sensing play the most important roles [1,2].

Recently, it was established that a regulatory protein encoded by the *rosR* gene is essential for the adaptation of *R. leguminosarum* to stress conditions, affecting the expression of many genes related to the synthesis of surface polysaccharides and cell-surface components, secretion of extracellular proteins, motility, and other cellular processes [3,4,5]. This gene encodes a 15.7 kDa global transcriptional regulator, which contains a Cys_2_-His_2_-type zinc finger motif responsible for binding to RosR-box motifs located in the promoter regions of the regulated genes [4,6]. The amino acid sequence of this motif revealed similarity to the Cys_2_-His_2_-type zinc finger motif, which is primarily found in DNA-binding proteins of eukaryotic origin [7,8,9]. In contrast to eukaryotic transcription factors (TFs), which typically contain tandem arrays of such Cys_2_-His_2_ zinc fingers, bacterial proteins, including the rhizobial RosR/MucR family, possess only one Cys_2_-His_2_-type zinc finger motif located in their C-terminal region.

RosR of *R. leguminosarum* shares significant similarity with Ros of *Rhizobium etli* and *Agrobacterium tumefaciens*, and with MucR of *Sinorhizobium meliloti* and *Sinorhizobium fredii* [10,11,12,13,14]. Mutations in *rosR* and *mucR* affect the function of both free-living rhizobia and during symbiosis with their host plants. In *S. meliloti*, MucR regulates the expression of genes involved in the synthesis of extracellular polysaccharides (EPS) (succinoglycan and galactoglucan), which are required for effective symbiosis with its macrosymbiont, alfalfa [15]. Thus, in this bacterium, inactivation of *mucR* abolishes the synthesis of succinoglycan and reduces cell motility, that in consequence, lead to disturbances in symbiosis [12,16,17]. Also in *S. fredii*, which shows a broad host range for nodulation, mutation in *mucR1* leads to a severe decrease in EPS biosynthesis, increased cell aggregation, and a drastic reduction in the nitrogen fixation capacity on *Glycine max* and *Lotus burttii* [13,18,19]. At least in this species, MucR1 is also required for the transcriptional activation of ion transporters engaged in bacterial nitrogen fixation in soybean nodules [14]. Similarly, the *Rhizobium etli* Ros protein is essential for EPS production, as well as for colonization and infection of its host plant, *Phaseolus* spp. [10]. In addition, a *rosR* mutant strain of *R. leguminosarum* produces a significantly smaller amount of EPS than the wild type, is more sensitive to surface-active compounds, exhibits decreased motility, and induces nodules with a high delay on clover roots, which are ineffective in nitrogen fixation [4,5].

Previously, we have established that the *R. leguminosarum rosR* open reading frame (ORF) possesses a 403 bp long upstream region containing several regulatory motifs [20,21]. Transcription of this gene was found to be driven by two promoters of different strengths: a distal strong promoter P1 and a much weaker proximal, P2 [6]. It was reported that P1 functions as the main promoter, and in addition to the −35 and −10 hexamers recognized by the RNA polymerase (RNAP) with σ^70^ subunit, it contains two other regulatory elements, an Upstream Promoter (UP) element [22,23] and a 3–4 bp long extension of the −10 element (TGn-extended −10 element), which in other bacteria are known to play a significant role in the initiation of transcription. We have confirmed that the AT-rich UP element located upstream of the −35 hexamer ensures a high level of expression of this gene from P1 [20]. The UP elements are recognized by the α subunits of the RNAP, whereas the 3–4 bp long extended −10 elements, which usually contain two nucleotides TG, located immediately upstream of the −10 hexamer are recognized by the σ subunit of this enzyme [23,24,25,26,27]. The expression of *rosR* driven by P2 is ~10-fold lower than that from P1. Downstream of the TGA stop codon of *rosR*, a *rho*-independent transcriptional terminator is located, which contains 12 nt long inverted repeats (IR) and forms a very stable stem structure.

It was reported that RosR recognizes and binds to a 22 bp RosR-box motif located downstream of *rosR* promoters and negatively regulates transcription of its own gene [6]. Moreover, binding sites for cAMP receptor protein CRP (cAMP-CRP) and Pho-boxes recognized by an activator PhoB, engaged in a positive regulation of *rosR* expression under carbon source and phosphate limitation, respectively, and a LysR motif (T-N_11_-A)_3_ recognized by proteins of the LysR family, were identified in the *rosR* upstream region [20,21]. In addition, a few motifs containing inverted repeats of different lengths (named IR1–IR6) were found in this region. Among them, IR5 contained the longest, 12 bp, inverted repeats. Up to now, only the role of IR4 in *rosR* expression was experimentally confirmed [21]. This motif was engaged in the negative regulation of *rosR* transcription, since its deletion or mutations resulted in considerably increased expression of the gene.

In this work, we examined the significance of several motifs identified in the upstream region of *R. leguminosarum* bv. *trifolii rosR* in the regulation of its own transcription. For this purpose, a set of *rosR-lacZ* transcriptional fusions, which contained mutations in these motifs was constructed. The levels of *rosR* transcription in different mutant variants in *R. leguminosarum* were established using both quantitative real-time PCR and β-galactosidase activity assays. The stability of wild type *rosR* transcripts and those containing mutations in IR motifs located in the 5′-untranslated region of this gene was determined using an RNA decay assay. In addition, the influence of chosen rhizobial regulatory proteins (a histidine kinase ChvG of a two-component regulatory system, and an activator CinR, and a represor PraR, both involved in quorum sensing) on the expression of *rosR* was studied.

## 2. Materials and Methods 

### 2.1. Bacterial Strains, Plasmids, and Growth Conditions

Bacterial strains and plasmids used in this study are listed in Table 1.

*R. leguminosarum* strains were cultured in 79CA medium with 1% glycerol as a carbon source at 28 °C with agitation (160 rpm) [38]. DF20 and VF39SM strains were grown in 79CA and Vincent’s minimal medium (VMM) supplemented with micronutrients and vitamin stock solution (2 mL L^−1^) [39] according to [40]. *Escherichia coli* strains were grown in Luria-Bertani (LB) medium at 37 °C [34]. When required, the media were supplemented with the appropriate antibiotics used at the following final concentrations: kanamycin, 40 μg mL^−1^, ampicillin, 100 μg mL^−1^, tetracycline, 10 μg mL^−1^, and nalidixic acid, 40 μg mL^−1^.

### 2.2. DNA Methods and Sequence Analysis

Standard molecular techniques, such as genomic and plasmid DNA isolation, restriction enzyme digestion, electrophoresis, cloning, and transformation were performed according to [34]. For PCR reactions, *Pfu* DNA Polymerase (Promega, Madison, WI, USA) or Ready-to-use RED-*Taq* DNA polymerase mix (Sigma-Aldrich, St. Louis, MO, USA) were used. Primers used in this work are listed in Table 2.

Sequencing was performed using the BigDye terminator cycle sequencing kit (Applied Biosystems, Foster City, CA, USA) and the ABI Prism 310 apparatus (Applied Biosystems). Database searchers were done with the FASTA and BLAST programs at the National Center for Biotechnology Information (https://www.ncbi.nlm.nih.gov/, Bethesda, MD, USA) and the European Bioinformatic Institute (https://www.ebi.ac.uk/, Hinxton, UK). Identification of the IR motifs in the *rosR* upstream region was performed using Malign and Fuzznuc programs [41,42,43]. RNA secondary structures and their stability were predicted using the Mfold program, version 2.3, and the default settings [44,45].

### 2.3. Construction of rosR-lacZ Transcriptional Fusions Containing Changed Sequences of Regulatory Motifs

The broad-host-range vector pMP220 carrying a promoter-less *lacZ* gene was used to construct plasmid fusions containing the *rosR* upstream region with mutated regulatory sequences [36]. For this purpose, plasmid pB31 containing the entire *rosR* gene and primers harboring changed sequences within the regulatory motifs (UP element, TGn-extended −10 element, IR1, IR2, IR3, IR5, IR6, and the RosR-box) and specific sequences for restriction enzymes were used (Table 2). Using primer pairs EP1/EB1, EP1/EB2, EP1/EB3, EP1/EB4, EP1/EB5, EP1/EB6, EP1/EB7, EP1/EB8, EP1/EB9, EP1/EB10, EP1/EB11, and EP1/EB12, 12 PCR products were obtained (PCR1 to PCR12), which encompassed the 5′-part of the *rosR* regulatory region. 12 DNA fragments encompassing the 3′-part of the *rosR* regulatory region were obtained using primer pairs BX13/RR1 (PCR13), BX14/RR1 (PCR14), BX15/RR1 (PCR15), BX16/RR1 (PCR16), BX17/RR1 (PCR17), BX18/RR1 (PCR18), BX19/RR1 (PCR19), BX20/RR1 (PCR20), BX21/RR1 (PCR21), BX22/RR1 (PCR22), BX23/RR1 (PCR23), and BX24/RR1 (PCR24). The first set of PCR products (PCR1–PCR8 and PCR11–PCR12) was digested with EcoRI and BamHI enzymes, whereas the second set of PCR products (PCR13–PCR20, PCR23, and PCR24) was digested with BamHI and XbaI. The exceptions were PCR9, PCR10, PCR21, and PCR22 fragments which were digested with PstI instead of BamHI. Next, PCR fragments (PCR1 and PCR13, PCR2 and PCR14, PCR3 and PCR15, PCR4 and PCR16, PCR5 and PCR17, PCR6 and PCR18, PCR7 and PCR19, PCR8 and PCR20, PCR9 and PCR21, PCR10 and PCR22, PCR11 and PCR23, and PCR12 and PCR24) were ligated together, and with pUC19 digested with EcoRI and XbaI, and then used to transform *E. coli* DH5α. The correctness of cloning was verified by PCR amplification, plasmid DNA isolation, restriction enzyme digestion, and sequencing. Subsequently, the resulting plasmids pPUC1–pPUC12 and the pMP220 vector were digested with EcoRI and XbaI, ligated, and introduced into DH5α strain. The correctness of the resulting plasmid constructs (named pM1 to pM12) was verified by colony PCR and enzyme digestions. The plasmids were then used to transform *E. coli* S17-1; the obtained derivatives were used as donor strains in bi-parental conjugation with *R. leguminosarum* bv. *trifolii* strain Rt24.2 as a recipient. The resulting Rt24.2 derivatives containing different *rosR-lacZ* transcriptional fusions were used for the determination of the amounts of different *rosR* transcript variants, as well as for the determination of β-galactosidase activities.

### 2.4. Construction of Plasmids Containing the Entire rosR Gene and Mutations in the Regulatory Motifs

To establish the stability of different *rosR* transcript variants, a set of plasmids containing the entire *rosR* gene and mutated sequences within the regulatory motifs was constructed using the expression vector pQE-31. For this purpose, pPUC7–pPUC12 plasmids and primers EP3/RR1 were used as templates for the amplification of the 5′-parts of the *rosR* upstream region (with mutations of IR5, IR6, and the RosR-box); pB31 and primers *rosR*-P/*rosR*-H were used for the amplification of the 3′-part of *rosR* (Table 2). A set of obtained PCR products (PCR25 to PCR30) was digested with EcoRI and PstI, whereas the PCR product obtained with primers *rosR*-P/*rosR*-H (PCR31) was digested with PstI and HindIII. Next, PCR fragments were ligated together (PCR25 and PCR31, PCR26 and PCR31, PCR27 and PCR31, PCR28 and PCR31, PCR29 and PCR31, and PCR30 and PCR31, accordingly) and the resulting products were subcloned into pQE-31 digested with EcoRI and HindIII. The different plasmids obtained (pQM7 to pQM12) were used to transform *E. coli* JM101. The control plasmid pQM1 was constructed using a 0.8 bp PCR fragment obtained with primers EP3 and *rosR*-H. The correctness of the resultant plasmids (pQM1 and pQM7–pQM12) was verified by PCR, enzyme digestions, and sequencing. *E. coli* JM101 derivatives containing these plasmids were used for the determinations of the life-time of different variants of *rosR* mRNAs.

### 2.5. RNA Isolation

To isolate total RNA from *R. leguminosarum* and *E. coli* strains, 5 mL of 24 h cultures in 79CA and LB, respectively, supplemented with the appropriate antibiotics were used. Bacterial pellets obtained after centrifugation of the cultures (12,000 × *g*, 10 min) were suspended in 1 mL of TRI-zol Reagent (Zymo Research, Irvine, CA, USA), vigorously mixed, and left for 5 min at room temperature. Next, the samples were centrifuged, and the supernatants were added to 1 mL of 95% ethanol, mixed, and subsequently applied to Zymo-Spin IIC columns from the Direct-Zol RNA MiniPrep kit (Zymo Research). Further steps of RNA purification were performed according to the manufacturer’s instructions. The concentration and purity of the isolated RNAs were determined spectrophotometrically using NanoDrop 2000 (Thermo Fisher Scientific, Waltham, MA, USA). Traces of DNA present in the RNA preparations were removed using the TURBO DNA-free kit containing DNase (Life Technologies, Waltham, MA, USA). The effectiveness of DNA elimination from RNA samples was checked by PCR using Ready-to-use RED-*Taq* DNA polymerase mix (Sigma-Aldrich) and primer pairs specific to rhizobial genes, *pssA* encoding a glucosyltransferase involved in the first step of EPS synthesis (*pssA*G1f/*pssA*2r) and *pssY* encoding a putative glycosyltransferase (*pssY*5f/*pssY*5r), or primers specific to the *E. coli* 16S ribosomal RNA (rRNA) gene (16Sec-F1/16Sec-R1).

### 2.6. Reverse Transcription and Quantitative Real-Time PCR

Real Time quantitative PCR (RT-qPCR) was used to determine the quantities and decay rates of the different variants of *rosR* transcripts. Complementary DNA (cDNA) was synthesized using 1 μg of total RNA and random hexamer primers (High-Capacity cDNA Reverse Transcription Kit, Life Technologies) as described earlier [46]. The following temperature profile was used: 25 °C (10 min), 37 °C (120 min), and 85 °C (5 min). Real Time quantitative PCRs were done using a Step One Plus PCR System and a Power SYBR Green PCR Master Mix (Thermo Fisher Scientific) in a 10-μL reaction volume, which contained gene-specific primers (Table 2). To determine the ratio of *rosR* cDNA to *recA* cDNA (encoding a protein required for homologous recombination) in *R. leguminosarum* samples, primers *rosR*4-Fw/*rosR*4-Rv specific to the 5′-region of the *rosR* ORF and primers *recA*2-Fw/*recA*2-Rv specific to the *recA* coding region were used. In the case of cDNAs obtained using RNAs isolated from *E. coli* strains, the same primers (*rosR*4-Fw and *rosR*4-Rv) for *rosR*, and primers 16SEc-F1/16SEc-R1 specific to 16S rRNA were employed. The temperature profile was as follows: denaturation at 95 °C for 1 min; followed by 40 cycles at 95 °C for 15 s, 58 °C for 30 s, and 60 °C for 30 s. Quantification cycle (C_q_) values for *rosR*-specific transcripts were normalized to *recA*, a housekeeping gene involved in homologous recombination in the *R. leguminosarum* background, or to the 16S rRNA gene in the *E. coli* background. The data shown are averages for triplicate biological replicates.

### 2.7. Determination of RNA Decay

The determination of *rosR* mRNA stability in *E. coli* cells was performed according to a method described by Pratte and Thiel [47,48], with minor modifications. For these analyses, *E. coli* JM101 derivatives containing plasmids pQM1 and pQM7–pQM12 were grown in 50 mL of LB supplemented with ampicillin at 37 °C (160 rpm) up to OD_600_ = 0.4 (optical density at 600 nm). Next, isopropyl-β-d-1-thiogalactopyranoside was added to a final concentration of 1 mM, and the cultures were grown for 1.5 h. After this time, 5 mL aliquots were collected from each culture (the 0 h time-point), 100 μg mL^−1^ rifampin was added to the cultures to inhibit new transcription, and the growth was continued for 1 h. Then, 10, 20, 30, 45, and 60 min after the addition of rifampin, 5 mL aliquots of the cultures were collected, quickly chilled on ice, harvested, and frozen. For each strain and time-point tested, three biological replicates were used. RNAs were isolated from these bacterial pellets using TRI-Reagent as described above. Reverse transcription and RT-qPCR were performed as described above (Section 2.6).

### 2.8. β-Galactosidase Assay

For this assay, *R. leguminosarum* and *E. coli* derivatives carrying plasmids with different transcriptional *rosR-lacZ* fusions were grown in a medium (79CA for *R. leguminosarum*, and LB for *E. coli*) supplemented with tetracycline for 24 h. The β-galactosidase activity was determined as described earlier using *orto*-nitrophenyl-β-d-galactopiranoside as a substrate [4]. 

### 2.9. Statistical Analysis

The statistical analyses of data were performed using the Student’s *t*-test and significant differences between the analyzed samples were established at *p* < 0.05.

## 3. Results

### 3.1. Directed-Mutagenesis and Functional Analysis of Regulatory Motifs Located in the rosR Upstream Region

**Figure 1:**
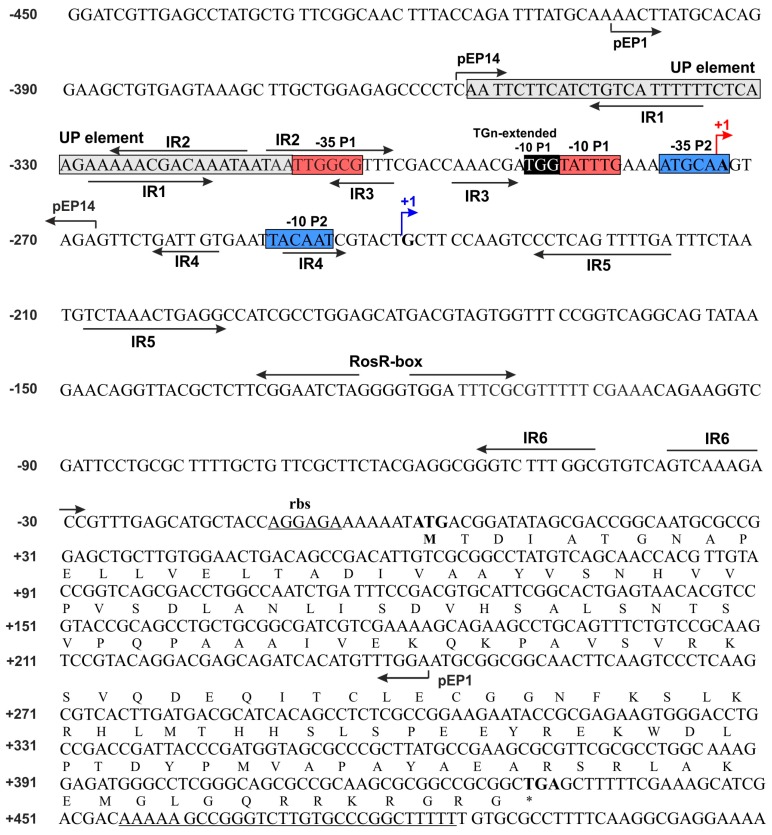
Nucleotide sequence of *Rhizobium leguminosarum* bv. *trifolii* Rt24.2 *rosR*, including its upstream region. The amino acid sequence of RosR is presented in the single letter code. P1 and P2 are promoters, whereas TS1 and TS2 are transcription start sites. The −35 and −10 hexamers of P1 and P2 promoters are marked by red and blue boxes, and TS1 and TS2 by red and blue arrows. The upstream promoter (UP) element and 3-4 bp long extension of the −10 hexamer (TGn-extended -10 element) are marked by grey and black boxes, respectively. The inverted repeats IR1 to IR6 and a palindromic sequence of the RosR-box are marked by inverted arrows. Over-line short arrows indicate the upstream and downstream endpoints of PCR fragments in the individual plasmid fusions (pEP1 and pEP14), respectively. pEP1 contains the *rosR* upstream region from −403 to +243 bp, whereas pEP14 harbors a promoter region from −357 to −268 bp. The ribosome-binding site (rbs) and a palindromic sequence of the *rho*-independent terminator are underlined.

Our previous studies showed that transcription of *rosR* is driven by two promoters, P1 and P2, each of them containing sequences highly similar to the eubacterial −35 and −10 sigma factor (σ^70^) binding sites (Figure 1) [6,20]. Among these, P1, containing two additional elements (UP and TGn-extended −10 elements), is the primary promoter responsible for ~90% of the *rosR* transcriptional activity. Moreover, *rosR* possesses a very long upstream region (403 bp), in which several putative regulatory motifs containing inverted repeats of different lengths (named IR1 to IR6) were identified. Among these, IR5 is the longest, containing 12 bp inverted repeats (Figure 1) [20,21]. Such long upstream regions are often described as target sites for the regulation of gene expression [49,50].

**Figure 2:**
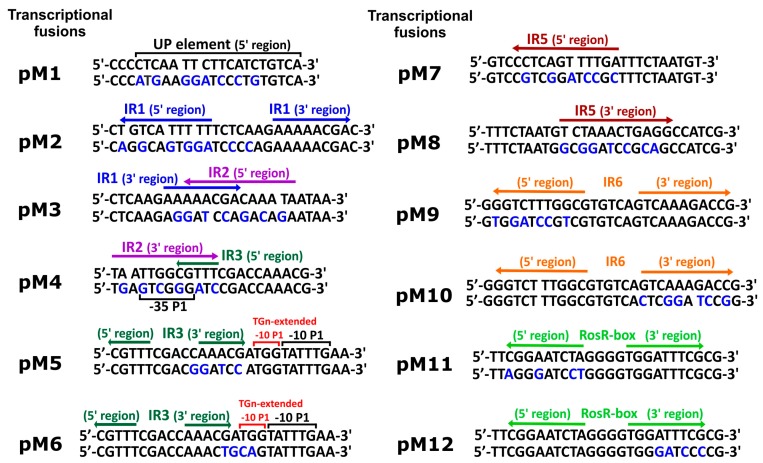
Nucleotide sequences of regulatory motifs identified in the upstream region of *R. leguminosarum* bv. *trifolii* Rt24.2 *rosR* and mutations introduced in these motifs (UP element, TGn-extended −10 element, and inverted repeats IR1, IR2, IR3, IR5, and IR6). Changed nucleotides in the sequences of the regulatory motifs on individual plasmids (pM1 to pM12) are designated by blue letters. UP and TGn-extended -10 elements are marked by black and red over-line lines, RosR-box is marked by light green arrows, whereas inverted repeats IR1, IR2, IR3, IR5, and IR6 are designated by blue, purple, dark green, dark red, and orange arrows, respectively.

In this study, we proceeded to establish the significance of individual regulatory motifs identified in the *rosR* upstream region for the expression of this gene. For this purpose, a set of transcriptional fusions between mutated versions of the *rosR* promoter (p*rosR*) and a promoter-less *lacZ* gene was constructed on the low-copy plasmid pMP220. Using oligonucleotide primers with changed sequences within the regulatory motifs, we obtained 12 plasmid constructs, named pM1 to pM12 (Figure 2). These p*rosR-lacZ* fusions harbored mutations within the UP element (pM1), 5′- and 3′-parts of the IR1 motif (pM2 and pM3), 3′-part of IR2 (pM4), 3′-part of IR3 (pM5), TGn-extended −10 element (pM6), 5′- and 3′-parts of IR5 (pM7 and pM8), 5′- and 3′-parts of IR6 (pM9 and pM10), and 5′- and 3′-parts of the RosR-box (pM11 and pM12) (Figure 2). Plasmid pEP1 containing the wild type p*rosR-lacZ* fusion was used as a control. 

**Figure 3:**
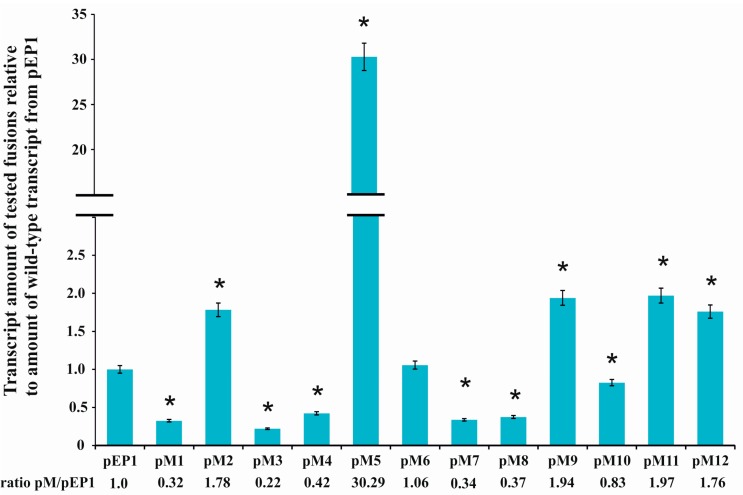
Quantitative Real Time PCR (RT-qPCR) analysis of *rosR* transcript levels using total RNA isolated from the *R. leguminosarum* bv. *trifolii* Rt24.2 derivatives containing pEP1 and pM1–pM12. Expression of *rosR* was normalized to the expression of *recA* ± standard deviation (SD). The ratio of the amount of transcripts carrying mutations within regulatory motifs to the amount of wild type transcript (pEP1) is given below the graph. Significant differences in the expression of *rosR* in the Rt24.2(pM) strains in relation to its expression in the control strain Rt24.2(pEP1) are indicated by an asterisk (* *p* < 0.05).

These plasmids were transferred by conjugation into *R. leguminosarum* bv. *trifolii* strain Rt24.2 in which subsequently the influence of mutations in the regulatory motifs on the *rosR* expression was studied. For this purpose, total RNA was isolated from the Rt24.2 derivatives containing pEP1 and pM1–pM12 plasmids, and used in RT-qPCR experiments (Figure 3). Comparative analysis of abundance of *rosR* transcripts in the strains containing different pM plasmids indicated that almost all changes introduced into the sequences of the analyzed regulatory motifs (with the exception of pM6) affected the level of *rosR* expression. Among these, the mutations present in pM2, pM5, pM9, pM11, and pM12 resulted in increased amounts of *rosR* transcripts in relation to that in the strain containing pEP1 (1.76-, 1.78-, 1.94-, 1.97-, and 30.29-fold for pM12, pM2, pM9, pM11, and pM5, respectively). This suggested that the motifs changed in pM2 (IR1), pM5 (IR3), and pM9 (IR6) were engaged in the negative regulation of *rosR* expression, and confirmed the role of the RosR-box (pM11 and pM12) in this type of regulation, as previously established [6].

On the other hand, significantly reduced amounts of *rosR* mRNAs were detected in Rt24.2 carrying the remaining plasmids (pM1, pM3, pM4, pM7, pM8, and pM10) in relation to the control Rt24.2(pEP1) (the ratio pM/pEP1 varied from 0.22 to 0.83, depending on the plasmid tested) (Figure 3). These data suggested that the UP element, and several IR motifs (IR2, IR5, and the 3′-part of IR6) participate in the positive regulation of the expression of this gene. Among the changes introduced in the examined motifs, the greatest effect was observed for the mutation in the 3′-part of IR3 located just upstream of the −10 P1 hexamer (~30-fold increase in *rosR* transcript amount in pM5 in relation to that of the control pEP1) (Figure 3).

**Figure 4:**
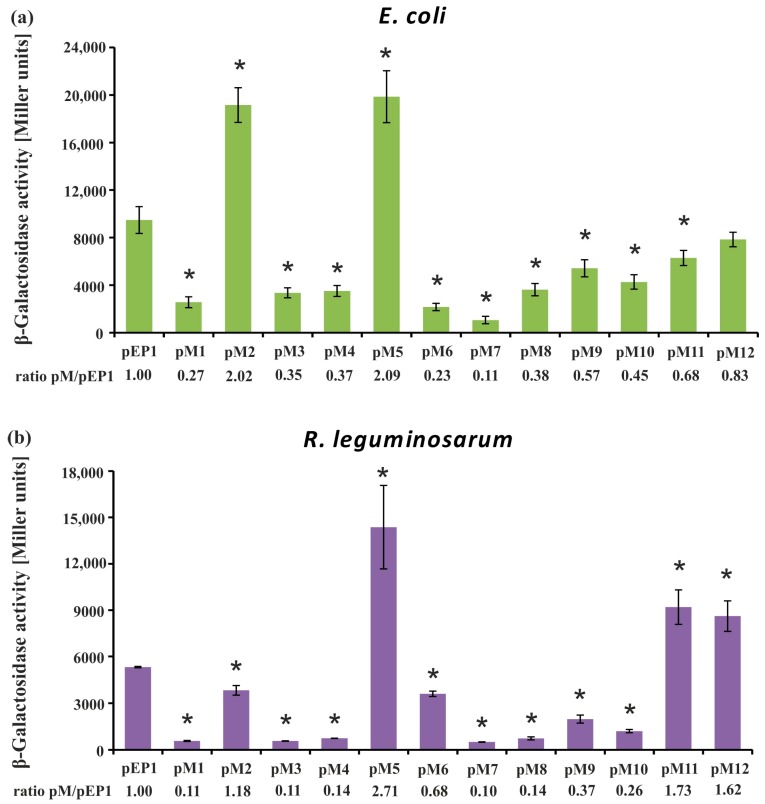
Determination of *rosR* expression in *Escherichia coli* DH5α (**a**) and *R. leguminosarum* bv. *trifolii* Rt24.2 (**b**) containing pEP1 and pM1–pM12 plasmids using the β-galactosidase activity assay. The ratio of β-galactosidase activity in the strains carrying pM plasmids to that in the strain carrying the control plasmid pEP1 is given below each graph. β-Galactosidase activity for pMP220 in *E. coli* was 12.5 ± 2.7, and in *R. leguminosarum* bv. *trifolii* it was 22.4 ± 3.6 Miller units. Significant differences in the expression level of *rosR* in the Rt24.2(pM) strains in relation to its expression in the control strain Rt24.2(pEP1), and in the *E. coli* DH5α(pM) in relation to *E. coli* DH5α(pEP1), are indicated by an asterisk (* *p* < 0.05).

In addition, the effect of mutations in the regulatory motifs on the level of *rosR* expression was examined using the β-galactosidase activity assay. These analyses were performed in both *R. leguminosarum* bv. *trifolii* and *E. coli* derivatives carrying plasmids pEP1 and pM1–pM12 (Figure 4).

Using this approach, we observed a tendency in *rosR* expression profiles in the two tested bacterial backgrounds that was similar to the RT-qPCR data, although values of β-galactosidase activity obtained in *E. coli*, which does not possess a RosR ortholog, were significantly higher than in *R. leguminosarum* (i.e., ~2-fold higher for the wild type pEP1 fusion). In general, β-galactosidase activity values obtained in both these strains carrying transcriptional fusions pM1, pM3, pM4, and pM6–pM10 were significantly lower than pEP1, indicating that the motifs, whose mutated sequences were harbored by these plasmids, are involved in the positive regulation of *rosR* expression. As noticed for pM1 in both tested backgrounds, mutation of the 5′-end of the UP element strongly reduced the transcriptional activity of this fusion, which confirmed the essential role of this promoter element in its activity. Moreover, the significance of IR2, which is located just upstream of the −35 P1 hexamer, in *rosR* expression was confirmed, since mutations within both parts of this motif highly reduced the level of *rosR* transcription (see pM3 and pM4) (Figure 3 and Figure 4). In this assay, a negative effect of the mutation in the TGn-extended −10 element on *rosR* expression (pM6) was observed; this effect was more pronounced in *E. coli* than in Rt24.2. Further, mutations in the motifs located downstream of transcription start sites TS1 and TS2 (IR5 and IR6) resulted in reduced *rosR* expression (plasmids pM7 to pM10), indicating their contribution to the determination of the optimal level of *rosR* expression. In contrast, mutations in both 5′- and 3′-parts of the RosR-box (pM11 and pM12) resulted, as expected, in elevated *rosR* transcription in the *R. leguminosarum* background. In these experiments, the strong positive effect of the mutation located within the 3′-end of IR3 (plasmid pM5) on the expression of this gene was also confirmed (Figure 4). This suggested that among the studied regulatory motifs, the IR3 sequence plays the most essential role in the determination of the *rosR* transcript levels. To establish whether the effects observed for pM5 and pM2 were caused by a generation of new promoter sequences as a result of the changes introduced into the motifs, in silico sequence analyses were performed. However, they did not reveal any novel promoter sequences (data not shown). 

All these data indicate that the analyzed motifs are involved in either positive or negative regulation of *rosR* transcription, suggesting that their collective activity might ensure the optimal level of its expression.

### 3.2. Determination of Secondary Structures of rosR Transcripts and Their Stability

Next, we performed additional analyses to establish whether the IR motifs located downstream of TS1 and TS2 (i.e., IR5, IR6, and the RosR-box) play a role in the generation of secondary structures in *rosR* mRNAs and their stability. Previously, we reported that two types of *rosR* transcripts of different lengths (766 nt and 733 nt long) are synthesized [6,21]. These transcripts contain 273 nt and 240 nt 5′-untranslated regions, respectively, in which IR5, the RosR-box, and IR6 are located. In the current study, we performed in silico sequence analysis of both the longer and shorter wild type *rosR* transcripts and their mutated variants with sequence changes within these motifs. We found that the shortening of the 766 nt transcript at the 5′-end did not dramatically influence its secondary structure and stability, which was confirmed by high and similar free energy values (ΔG) (*−*348.89 for the 766 nt long and *−*334.87 kcal/mol for the 733 nt long transcript, respectively) (Table 3).

In silico predictions revealed that the tested motifs were involved in the formation of secondary structures in *rosR* transcripts (data not shown). However, although mutations introduced into IR5, the RosR-box, and IR6 affected the formation of secondary structures in these transcripts, they only slightly decreased transcript stability. In fact, the greatest changes in the absolute ΔG values were from −348.89 and −334.87 kcal/mol, for the wild type transcripts, to −334.05 and −330.29 kcal/mol, in the case of mutations in the 3′-part of IR5 (Table 3). Alterations of the 3′-part of the RosR-box did not affect ΔG values of the *rosR* transcripts.

**Figure 5:**
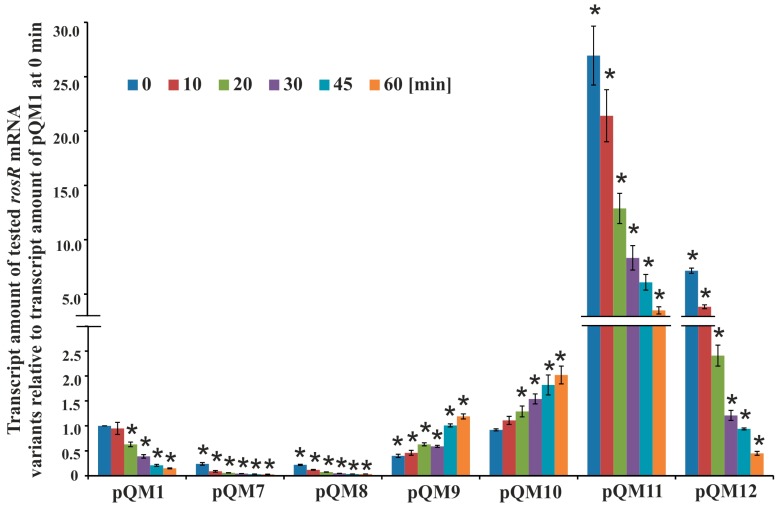
The stability of different variants of *rosR* transcripts. Transcript stability was determined by measuring transcript amount by RT-qPCR, using RNAs isolated from *E. coli* derivatives carrying pQM1 and pQM7–pQM12 plasmids with mutations in the IR5, IR6, and RosR-box motifs. Bacteria were collected 0, 10, 20, 30, 45, and 60 min after the addition of rifampin. The data shown are averages from three biological replicates ± SD. The statistically significant differences between the amount of different variants of *rosR* transcripts in relation to the amount of the wild type transcript in the control strain *E. coli*(pQM1) at the same time-point are indicated by an asterisk (* *p* < 0.05).

To experimentally confirm the role of individual IR motifs in the stability of *rosR* transcripts, a set of plasmids containing the entire *rosR* ORF with either the wild-type or altered (by mutations in IR5, IR6, or the RosR-box) upstream region was constructed using the pQE-31 vector (pQM1 and pQM7–pQM12). To avoid the influence of other rhizobial regulators than RosR on *rosR* expression, we examined the stability of these transcripts in an *E. coli* background. The analysis was performed by quantifying mRNA after the addition of rifampin, which inhibits the initiation but not elongation of transcription involving RNAP [51]. In this experiment, the strains containing the complete wild type *rosR* sequence (plasmid pQM1) or mutations in the 5′- and 3′-parts of IR5 (plasmids pQM7 and pQM8), 5′- and 3′-parts of IR6 (pQM9 and pQM10), and 5′- and 3′-parts of the RosR-box (pQM11 and pQM12) were used. The abundance of different variants of *rosR* mRNAs was measured by RT-qPCR at various time-points after the addition of rifampin (from 0 to 60 min). The data presented in Figure 5 were normalized to the amount of the wild type transcript synthesized in *E. coli* (pQM1) at time 0 min.

In general, the wild type *rosR* transcript proved to be very stable and its degradation was very slow, which confirmed its long half-life (25.2 ± 4.2 min); 95% of its amount at time 0 min was detected after 10 min, and 15% after 60 min from the transcription inhibition. However, the patterns of degradation of the mutated versions of *rosR* transcripts were essentially different from that of the wild type. Mutations in both 5′- and 3′-parts of IR5 and in the 5′-part of IR6 negatively affected *rosR* transcription, as suggested by the very low transcript amounts found in *E. coli*(pQM7), *E. coli*(pQM8), and *E. coli*(pQM9) strains at 0 min in relation to control strain *E. coli*(pQM1) (Figure 5). This difference was about 4-fold for transcripts from *E. coli*(pQM7) and *E. coli*(pQM8), and 2.5-fold for that from *E. coli*(pQM9). Surprisingly, the transcripts containing mutations in IR5 differed in their stability with respect to those having mutations in IR6. The first ones decayed very quickly and their half-life was 8.2 ± 1.7 min (pQM7) and 11.3 ± 3.2 min (pQM8), respectively. In contrast, the transcripts with mutated 5′- or 3′-parts of IR6 (pQM9 and pQM10) behaved similarly and were very stable. They did not degrade or even, their amounts slightly increased during the time of the experiment, which could be explained in part by the fact that RNA polymerase molecules that were already transcribing at the time of rifampin addition would complete their transcripts, as it has been described previously for the ATPase operon of *Prochlorococcus* MED4 [52].

In contrast, we observed another effect in the case of mutations within the RosR-box (Figure 5). Significantly higher amounts of *rosR* transcripts in *E. coli*(pQM11) and *E. coli*(pQM12) strains were found in relation to the control *E. coli*(pQM1) at 0 min. This indicated that the introduced changes positively affected the level of *rosR* transcription in this genetic background. Moreover, this finding suggested that the *R. leguminosarum* RosR protein was effectively synthesized in *E. coli* from pQM plasmids and bound to the wild type sequence of the RosR-box, strongly repressing *rosR* transcription. This was confirmed by pronounced differences in the abundance of *rosR* transcripts in *E. coli*(pQM11) and *E. coli*(pQM12) in comparison with *E. coli*(pQM1). However, mutations within the RosR-box did not affect the stability of the synthesized transcripts as pronouncedly as mutations in IR5, as indicated by the half-lives (for transcripts containing changed 5′- and 3′-regions of the RosR-box these were 17.8 ± 3.0 min (pQM11) and 12.5 ± 4.1 min (pQM12), respectively).

In summary, the presented data confirmed that the IR5 and IR6 motifs play an important role in the determination of the *rosR* transcription level and/or stabilization of the synthesized transcript. These motifs are engaged in the formation of secondary structures of *rosR* RNA and their stability, that in consequence, affects transcript life-time in bacterial cells.

### 3.3. The Influence of CinR, PraR, and ChvG on the Expression of rosR

In addition, we decided to study whether some rhizobial regulators could affect the expression of *rosR*. For this experiment, we selected proteins that play an important role in quorum sensing (transcriptional regulators CinR and PraR) and rhizobial signaling (histidine kinase ChvG of a two-component regulatory system), since mutations in genes encoding these proteins lead to effects that are similar to those observed for the *rosR* mutant (i.e., changes in biofilm formation, cell-surface properties, and the synthesis of surface polysaccharides) [4,29,31,33,53].

**Figure 6:**
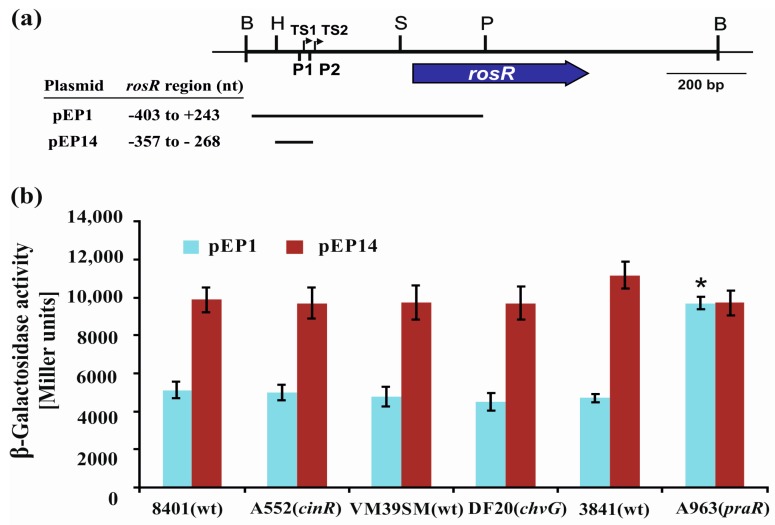
(**a**) A physical and genetic map of plasmid pB31 carrying *R. leguminosarum rosR*. The blue arrow below the map shows the direction of *rosR* transcription. P1 and P2 are promoter sequences, whereas TS1 and TS2 are transcription start sites. Lengths of the *rosR* upstream region fragments in pEP1 and pEP14 plasmids are shown as horizontal lines. (**b**) Transcriptional activity of *rosR* assayed in wild type *R. leguminosarum* strains (8401, VM39SM, and 3841) and their derivatives with mutations in *cinR*, encoding a positive regulator involved in quorum sensing (A552), *chvG*, encoding a histidine kinase ChvG of a two-component regulatory system (DF20), and *praR*, encoding a repressor related to quorum sensing (A963), that carried pEP1 and pEP14 plasmids with different fragments of the *rosR* upstream region fused with a promoter-less *lacZ* gene. β-Galactosidase activities (presented as Miller units) for the tested plasmid pEP fusions are given as the averages from three independent experiments ± SD. Significant differences in *rosR* expression between 3841 and A963 (*praR*) for the individual fusions tested are indicated by an asterisk (* *p* < 0.05). B: BamHI; H: HindIII; P: PstI; S: SphI.

First, plasmid fusions pEP1 and pEP14 (Figure 6a) were transferred to *cinR*, *praR*, and *chvG* mutants and their wild type strains, and β-galactosidase activities were assayed (Figure 6b). The pEP1 plasmid contained the entire *rosR* upstream region (from −403 to +243 bp), whereas pEP14 harbored a shorter promoter region encompassing only the UP element and the P1 promoter (from −357 to −268 bp) (Figure 1 and Figure 6a). The levels of *rosR* expression in the tested strains carrying pEP1 and pEP14 were high and similar to those described previously for the *R. leguminosarum* strain Rt24.2 [20]. In the three wild type strains tested, β-galactosidase activities provided by pEP14 were higher than those obtained in the presence of pEP1. When the individual plasmid fusions were analyzed, no differences were observed in the β-galactosidase activity levels in the *chvG* mutant DF20 and its wild type strain VF39SM, and in the *cinR* mutant A552 and its wild type strain 8401 (Figure 6b). This indicated that CinR or ChvG do not affect *rosR* expression. In contrast, a significant difference in β-galactosidase activities was noted for the pEP1 fusion in the *praR* mutant A963 and its wild type strain 3841 (Figure 6b), suggesting that PraR could affect *rosR* expression. Frederix and others [29] have previously characterized a CAAC-N_5_-GTTG consensus recognized by PraR. We identified a sequence motif CAAGTAGAGTTC in the *rosR* upstream region (from −275 to −263 nt), which showed a similarity to PraR-binding site (nucleotides identical to those in the consensus are underlined). This sequence, present in pEP1, was truncated in pEP14.

## 4. Discussion

In this study, we showed that transcription of the *R. leguminosarum rosR* gene undergoes a complex regulation, in which several *cis*-regulatory elements and a previously unidentified *trans*-acting factor are engaged. Mutational analysis of regulatory motifs identified in the *rosR* upstream region confirmed a significant role of some of these elements in the modulation of transcription and/or transcript stability of this gene. In general, transcription of *rosR* was high (see transcriptional activity of *rosR* in the wild type fusion pEP1 in Figure 4 and Figure 6), and the main promoter P1 was responsible for this effect (see transcriptional activity of *rosR* provided by UP and P1 in pEP14 in Figure 6).

According to the definition of Gottesman, RosR belongs to global regulators, on the basis of its pleiotropic phenotype and ability to regulate operons associated with different metabolic pathways [54]. In fact, RosR directly or indirectly affects a large group of *R. leguminosarum* genes (1106), with the majority of them negatively regulated, indicating that RosR functions mainly as a repressor. These genes are associated with the synthesis of cell-surface components, envelope biogenesis, motility, transport and metabolism of carbohydrates and nitrogen sources, and other cellular processes, such as signal transduction and transcription regulation [4,55]. The RosR-box motifs identified in the promoter regions of genes directly regulated by RosR shared a low similarity with the RosR-box consensus. It is well-known that the regulatory effect of individual TFs depends on their concentration and affinity to binding sites; to function, weak sites (i.e., sites with a low sequence similarity to the consensus) require high concentrations of TFs, whereas strong binding sites work with a lower amount of TFs [56]. In contrast to local TFs that tend to have high-affinity sites, global TFs are less specific, bind to a larger collection of sites, and therefore, must be expressed at higher levels [57,58]. This is in agreement with our observation of a high level of *rosR* transcription. This effect was associated with the activity of the P1 promoter, which in addition to the two core elements (−35 and −10 hexamers) recognized by RNAP with σ^70^ subunit, contains two *cis*-regulatory elements (UP and TGn-extended −10) that are present only in a small number of bacterial promoters [59]. Three domains of RNAP σ^70^ are responsible for recognizing and binding the −10 hexamer (domain 2), −10 extension (domain 3), and −35 hexamer (domain 4), whereas C-terminal domains of the two RNAP α subunits can interact with the UP element located upstream of the −35 region. UP elements characterized in *E. coli* promoters are ~30 bp A/T-rich sequences that contain two distal and proximal regions, and their presence can stimulate transcription up to 300-fold, depending on the gene studied [23,24,25,60]. Several studies reported that UP elements enhance the transcription of downstream genes, although so far they have been described only for a small number of bacterial promoters, mainly in *E. coli* (such as P1 of the rRNA *rrnB* and the *guaB* promoter required for the *de novo* synthesis of GMP) but also in other bacteria, such as *Bacillus subtilis* [23,24,25,61,62,63,64]. Moreover, the presence of UP in promoters served by RNAP containing σ^70^ might reduce their dependence on the consensus of the −10 or −35 elements [27]. To the best of our knowledge, *R. leguminosarum rosR* P1 is the first described example of a rhizobial promoter containing UP and TGn-extended −10 elements [23,24,25,26,27]. 

In this study, we performed mutational analyses of 12 sequence motifs located in the *rosR* upstream region, including the UP and TGn-extended −10 elements, to examine their role in the transcription of this gene. *rosR* expression is driven by two promoters, the strong P1 promoter and the very weak P2 promoter. The fact that the majority of the motifs studied are located immediately upstream or even inside P1 most probably means that their mutations mainly affect P1-driven *rosR* expression. Based on the results obtained for different variants of this region (plasmids pM1–pM12), we observed that the UP present in the *rosR* P1 promoter was longer (46 nt) than those characterized in *E. coli* (30 nt), and that it was functional in both *R. leguminosarum* and *E. coli* backgrounds, since mutations of both the 5′- and 3′-regions of this element (pM1 and pM3, respectively) considerably reduced *rosR* transcription in both bacteria (3-fold in *E. coli* and 10-fold in *R. leguminosarum*) (Figure 4). This confirmed that the UP element plays an essential role in the stimulation of *rosR* expression from the P1 promoter. However, alteration of the 5′-region of the IR1 element located in UP (pM2) had an opposite effect, resulting in a ~2-fold increase of *rosR* transcription in both tested bacteria (Figure 3 and Figure 4). To elucidate whether the changes introduced in the sequence of this motif might have resulted in the appearance of an additional promoter sequence, bioinformatics analyses of the *rosR* upstream region containing the changed IR1 5′-end were performed; the analyses did not reveal any such new sequences. Therefore, we propose that the alteration of this sequence may have contributed to a stronger interaction of RNAP α subunits with this regulatory region or may be connected with the loss of either a repressor-binding site or a silencing region, which would normally attenuate *rosR* expression from P1. In contrast, mutation of IR2 (pM3 and pM4) had a negative effect on *rosR* transcription. Taken together, these results indicated that not only the A/T-rich composition of UP, but also its local structural organization and sequence might be important for transcription initiation, and that IR1 and IR2 exert a negative and positive effect on *rosR* expression, respectively. Surprisingly, we did not observe a pronounced effect of the mutation in the TGn-extended −10 element (plasmid pM6) (Figure 3 and Figure 4). Based on the approach used, only a weak negative effect was observed in *R. leguminosarum* using β-galactosidase assay and no effect in RT-qPCR. The effect of this mutation was more pronounced in *E. coli* (a 4-fold decrease of *rosR* transcription in pM6 in relation to the wild type pEP1). These data indicated that the 3 bp long −10 extension does not play such an important role in the transcription of this gene in *R. leguminosarum*, as described for some *E. coli* genes [58,59,60]. However, a mutation in the IR3 3′-region located just upstream of the −10 extension (pM5) resulted in a strong positive effect in both genetic backgrounds tested, confirming that IR3 also plays a negative role in *rosR* transcription. Further, in this case the performed in silico analyses excluded the occurrence of a new, additional promoter in this region. Thus, one of the possible explanations of this phenomenon might be the loss of a site recognized by a repressor, which would be conserved between α- and γ-Proteobacteria. Moreover, the possibility of the loss of a silencing region that normally attenuates *rosR* expression from P1 to allow regulation via the downstream promoter P2 cannot be excluded. Another possibility is that the observed elevated *rosR* transcription from pM5 is associated with a putative interaction of RNAP σ^70^ domains with the IR3 3′-region, which might be stronger in the case of its mutated version than in the wild type sequence. Our results suggest that, apart from the −10 extension, a short sequence (7 bp) adjacent to this motif might be engaged in the RNAP σ^70^–*rosR* promoter binding, and that at least in some cases, e.g., *rosR*, the −10 extension might be longer than 3 bp. The fact that the mutations in pM5 and pM2 similarly affected *rosR* expression in both *R. leguminosarum* and *E. coli* suggests that such element(s) may be conserved in both bacteria. Moreover, mutations introduced in IR1–IR3 most probably do not affect the *rosR* mRNA stability since these motifs are located upstream of the transcription start sites. Similarly, a highly complex mechanism of action was detected for other bacterial global regulatory proteins. For example, fumarate-nitrate reduction regulator Fnr was found to play a dual role in the regulation of *arcA*, which encodes an aerobic respiratory control protein in *E. coli*, depending on the growth conditions tested (anaerobiosis/aerobiosis) [65,66]. This gene possesses a long non-coding upstream region (530 bp) containing five promoters recognized by RNAPσ^70^ and Fnr can function either as an activator from a distal arcAp1 promoter or as a repressor from arcAp3 promoter by binding to the same Fnr-box sequence in this region (−284 bp).

In this study, we also established the role of the IR5 and IR6 motifs, and the RosR-box, located downstream of the transcription start sites TS1 and TS2, in the formation and stabilization of *rosR* RNA secondary structures. In silico sequence analysis of the wild type and mutated versions of *rosR* transcripts indicated that these sequence motifs impacted the RNA secondary structure. Based on the results obtained using plasmids pM7–pM12, we experimentally confirmed that mutations of both 5′- and 3′-parts of IR5 and IR6 negatively affected *rosR* transcription in *R. leguminosarum* and *E. coli* (Figure 3 and Figure 4). The greatest reduction was observed for mutations within IR5 (in the rhizobial background). RNA decay analysis performed in *E. coli* using plasmids pQM1 and pQM7–pQM12 confirmed that wild type *rosR* transcripts were very stable in bacterial cells, and that IR5 located at the 5′-end of the *rosR* mRNAs plays the most essential role in their synthesis and protection against degradation (Figure 5). In contrast, the IR6 motif decreased the stability of the transcript since its inactivation increased the half-life of *rosR* mRNA. Thus, our data indicated that IR5 and IR6 play opposite roles in the stability of *rosR* transcripts. Moreover, considerably higher amounts of *rosR* transcripts were detected in the case of plasmids pQM11 and pQM12, which harbor mutations within the RosR-box, than in the wild type pQM1. This suggested that (i) RosR was effectively synthesized in *E. coli*, and (ii) this rhizobial protein was functional in *E. coli* (i.e., recognized the wild type RosR-box and negatively regulated the transcription of its own gene). However, this motif did not play such an important role in the stability of *rosR* transcripts as the IR5 and IR6 motifs.

As reported by Pratte and Thiel, for several genes in *nif* cluster (*nifB1*, *nifS1*, *nifH1*, *nifE1*, *nifD1*, *nifU1*, *nifK1*, *nifN1*, *nifX1*, *hesA1*, and *fdxH1*) encoding proteins involved in nitrogen fixation in the cyanobacterium *Anabaena variabilis* [47], the half-lives of individual *nif* mRNAs are very different; from as high as 33 min (for *nifH1*) and ~20 min (for *nifD1*, *hesA1* required for efficient nitrogen fixation, and *fdxH1* encoding the [2Fe-2S] ferredoxin that is an electron donor to nitrogenase, a key enzyme in nitrogen fixation), to as low as ~8 min (*nifE1* and *nifU1*). In comparison to these data, *R. leguminosarum rosR* transcript belongs to those characterized by high life-time. These authors also showed that the degradation patterns of these mRNAs were strictly different, confirming that this is a specific property of individual gene’s mRNA. The structural organization of promoters of these *nif* genes and their transcript stability proved to be important for their abundance and life-time in the cell. Similarly to our findings for IR5, they reported that the stem-loop structure upstream of *nifH1* controlled the abundance of *nifH1* mRNA through transcript processing and stabilization [48]. Stem-loops stabilize transcripts when they are at the extreme 5′-end of the transcript, since these double-stranded structures prevent mRNA recognition by 5′-exonuclease [67,68].

In this work, we also examined whether some regulatory proteins which play an important role in processes such as quorum sensing (CinR and PraR) or signaling (ChvG) in rhizobia, affect *rosR* transcription [29,31,33,53]. Based on the results obtained for the wild type *R. leguminosarum* and its *chvG*, *cinR*, and *praR* mutant strains, we confirmed that PraR may act as a *trans*-regulatory factor able to repress *rosR* expression (Figure 6). In fact, a sequence with a high similarity to the PraR-binding site was identified downstream of the P1 -10 motif. However, further studies are required to effectively prove a direct effect of PraR on the *R. leguminosarum rosR* expression. Inhibition of *rosR* expression by PraR in *E. coli*, which lacks a PraR ortholog, might provide evidence for the direct interaction. We plan to perform these types of studies in the near future. Recently, Frederix and others [53] characterized a consensus sequence recognized by PraR, and reported that this TF effectively binds to the promoters of *rapA2*, *rapB*, and *rapC* (which encode adhesins), *plyB*, *rosR*, and its own promoter, and negatively regulates transcription of these genes. PraR is important for biofilm formation both in vitro and on plant roots, i.e., during a step that precedes the initiation of rhizobial infection of legume roots. Mutation in *praR* enhanced root biofilms and improved nodulation competitiveness of the bacterium, most probably by increasing the expression of genes coding for proteins involved in bacterial attachment to host root surfaces. All these data indicate that RosR and PraR are important elements of the rhizobial regulatory network, which is required by the bacteria to constantly monitor the extracellular physiological conditions and to respond by modifying gene expression pattern to adjust their growth [69,70].

## 5. Conclusions

Bacterial TFs play an important role in the genetic regulation of transcription in response to external and internal cellular stimuli. In *R. leguminosarum*, the TF encoded by *rosR* is a global regulator that plays an essential role in this regulatory network. Here, we reported that transcription of *rosR* undergoes a complex regulation, with the involvement of several *cis*-acting regulatory elements identified in its upstream region and the *trans*-acting repressor PraR. Among them, apart from the −35 and −10 boxes of the distal P1 promoter, the most essential elements are the A/T-rich UP element located upstream of the P1 −35 hexamer, the IR3 3′-region located upstream of the −10 extension, as well as the inverted repeats IR5 and IR6. These elements are involved in the modulation of the level of *rosR* transcription and/or the formation of secondary structures and the stability of *rosR* transcripts, which, consequently affect their life-time in the cell. Most probably, such a complex arrangement of both negative and positive *cis*-acting elements (even overlapping) in the upstream region of this gene is linked to the requirement for fine-tuning of the regulation of the expression of global regulatory proteins, such as RosR.

## Figures and Tables

**Table 1:** Bacterial strains and plasmids used in this study.

Strains and Plasmids	Characteristics	Source or Reference
*Rhizobium leguminosarum*		
Rt24.2	Wild type, Rif^r^, Nx^r^	[6]
3841	Wild type, Sm^r^	[28]
A963	3841 *praR*::Tn*5*, Km^r^	[29]
8401	Wild type, Sm^r^	[30]
A552	8401 *cinR1*::Tn*5*, Km^r^	[31]
VF39SM	Wild type, Sm^r^	[32]
DF20	VF39SM *chvG*::Tn*5*, Km^r^	[33]
*Escherichia coli*		
DH5α	*supE*44 Δ*lac*U169 (φ80 *lacZ*Δ M15) *hsdR*17 *recA*1*endA*1*gyrA*96 *thi*-1 *relA*1	[34]
S17-1	294, *thi*, RP4-2-Tc::Mu-Km::Tn*7*	[35]
JM101	*supE thi-1* Δ(*lac*-*proAB*) F′ [*traD36 proAB*^+^ *lacI*^q^ *lacZ*Δ*M15*]	[34]
Plasmids		
pUC19	Cloning and sequencing vector, Ap^r^	[34]
pMP220	IncP, *mob*, promoterless *lacZ*, Tc^r^	[36]
pQE-31	*ori* ColE1, expression vector, Ap^r^	[37]
pB31	pUC19 carrying 1.17 kb BamHI fragment with Rt24.2 *rosR*	[6]
pPUC1	pUC19 carrying 647 bp EcoRI-XbaI fragment (*rosR* region from −403 to +243 bp) with a changed sequence in the 5′-part of the UP element	This work
pPUC2	pUC19 carrying 647 bp EcoRI-XbaI fragment (*rosR* region from −403 to +243 bp) with a changed sequence in the 3′-part of the UP element	This work
pPUC3	pUC19 carrying 647 bp EcoRI-XbaI fragment (*rosR* region from −403 to +243 bp) with a changed sequence in the IR1 3′-part and IR2 5′-part	This work
pPUC4	pUC19 carrying 647 bp EcoRI-XbaI fragment (*rosR* region from −403 to +243 bp) with a changed sequence in the P1-35, IR2 3′-part, and IR3 5′-part	This work
pPUC5	pUC19 carrying 647 bp EcoRI-XbaI fragment (*rosR* region from −403 to +243 bp) with a changed sequence in the 3′-part of IR3	This work
pPUC6	pUC19 carrying 647 bp EcoRI-XbaI fragment (*rosR* region from −403 to +243 bp) with a changed sequence in the TGN-extended −10 motif	This work
pPUC7	pUC19 carrying 647 bp EcoRI-XbaI fragment (*rosR* region from −403 to +243 bp) with a changed sequence in the 5′-part of IR5	This work
pPUC8	pUC19 carrying 647 bp EcoRI-XbaI fragment (*rosR* region from −403 to +243 bp) with a changed sequence in the 3′-part of IR5	This work
pPUC9	pUC19 carrying 647 bp EcoRI-XbaI fragment (*rosR* region from −403 to +243 bp) with a changed sequence in the 5′- part of IR6	This work
pPUC10	pUC19 carrying 647 bp EcoRI-XbaI fragment (*rosR* region from −403 to +243 bp) with a changed sequence in the 3′-part of IR6	This work
pPUC11	pUC19 carrying 647 bp EcoRI-XbaI fragment (*rosR* region from −403 to +243 bp) with a changed sequence in the 5′-part of the RosR-box	This work
pPUC12	pUC19 carrying 647 bp EcoRI-XbaI fragment (*rosR* region from −403 to +243 bp) with a changed sequence in the 3′-part of the RosR-box	This work
pEP1	pMP220 carrying the −403 to +243 bp *rosR* upstream region	[6]
pEP14	pMP220 carrying the −358 to −268 bp *rosR* upstream region	[20]
pM1	pMP220 carrying 647 bp EcoRI-XbaI fragment of pPUC1	This work
pM2	pMP220 carrying 647 bp EcoRI-XbaI fragment of pPUC2	This work
pM3	pMP220 carrying 647 bp EcoRI-XbaI fragment of pPUC3	This work
pM4	pMP220 carrying 647 bp EcoRI-XbaI fragment of pPUC4	This work
pM5	pMP220 carrying 647 bp EcoRI-XbaI fragment of pPUC5	This work
pM6	pMP220 carrying 647 bp EcoRI-XbaI fragment of pPUC6	This work
pM7	pMP220 carrying 647 bp EcoRI-XbaI fragment of pPUC7	This work
pM8	pMP220 carrying 647 bp EcoRI-XbaI fragment of pPUC8	This work
pM9	pMP220 carrying 647 bp EcoRI-XbaI fragment of pPUC9	This work
pM10	pMP220 carrying 647 bp EcoRI-XbaI fragment of pPUC10	This work
pM11	pMP220 carrying 647 bp EcoRI-XbaI fragment of pPUC11	This work
pM12	pMP220 carrying 647 bp EcoRI-XbaI fragment of pPUC12	This work
pQM1	pQE-31 carrying 0.8 kb EcoRI-HindIII fragment with the wild-type *rosR*	This work
pQM7	pQE-31 carrying 0.8 kb EcoRI-HindIII fragment with *rosR* mutated in IR5 5′	This work
pQM8	pQE-31 carrying 0.8 kb EcoRI-HindIII fragment with *rosR* mutated in IR5 3′	This work
pQM9	pQE-31 carrying 0.8 kb EcoRI-HindIII fragment with *rosR* mutated in IR6 5′	This work
pQM10	pQE-31 carrying 0.8 kb EcoRI-HindIII fragment with *rosR* mutated in IR6 3′	This work
pQM11	pQE-31 carrying 0.8 kb EcoRI-HindIII fragment with *rosR* mutated in the 5′-part of the RosR-box	This work
pQM12	pQE-31 carrying 0.8 kb EcoRI-HindIII fragment with *rosR* mutated in the 3′-part of the RosR-box	This work

Nx^r^, nalidixic acid resistance; Rif^r^, rifampicin resistance; Tc^r^, tetracycline resistance; Ap^r^, ampicillin resistance; Km^r^, kanamycin resistance; Sm^r^, streptomycin resistance; *Ori*, origin of replication; IncP, plasmid from the incompatibility group IncP; *mob*, mobilization operons; UP, upstream promoter.

**Table 2:** Oligonucleotide primers used in this study.

Primer	Sequence (5′–3′) ^1^	Source or Reference
EB1	TGACAGATGGATCCTTCATGGGCT	This work
EB2	CCCTCAAGGATCCCTGTGTCATTT	This work
EB3	TTCTTGGGATCCACTGCCTGATGAA	This work
EB4	GTCATTGGATCCCCAGAAAAACGA	This work
EB5	ATTTGTGGATCCTCGTGAGAAAAAA	This work
EB6	CTCAAGAGGATCCAGACAGAATAA	This work
EB7	TTGGTCGGATCCCGACTCATTATTT	This work
EB8	AATTGGGGATCCGACCAAACGAT	This work
EB9	TCAAATACTGCAGTTTGGTCGAAACG	This work
EB10	GACCAAACTGCAGTATTTGAAAATGCAAG	This work
EB11	TACCATGGATCCGTCGAAACGCCA	This work
EB12	GTTTCGACGGATCCATGGTATTTGAA	This work
BX13	TAGAAATCGGATCCGACGGACTTGG	This work
BX14	GTCCCTCGGATCCGCTTTCTAATGT	This work
BX15	TGGCCTCGGATCCGCCATTAGAAA	This work
BX16	TAATGTCGGATCCGCAGCCATCG	This work
BX17	ACCCCTGGATCCCTAAGAGCGTAA	This work
BX18	TCTTCGGGATCCTGGGGTGGATTT	This work
BX19	AAACGCGGATCCCTCCCCTAGATT	This work
BX20	GGGTGGGATCCCCGTTTTTCGAAA	This work
BX21	AAACGGGATCCGAGTGACACGCCA	This work
BX22	TCAGTCGGATCCGGTTTGAGCATG	This work
BX23	ACGCCGGATCCACGCCTCGTAGAA	This work
BX24	AGGCGGGGATCCGTCGTGTCAGT	This work
EP1	ATGCAAGAATTCTGCACAGGAAGC	[6]
RR1	CGCATTCTAGACATGTGATCTGCT	[6]
EP3	GGTATTTGGAATTCCAAGTAGAGTTCT	[6]
*rosR*-P	AAAGCAGAAGCCTGCAGTTTCTGT	This work
*rosR*-H	TCCTGACAAGCTTCATCGAGATTA	This work
*rosR*4-Fw	GCGACCTGGCCAATCTGATTTC	This work
*rosR*4-Rv	CTGCAGGCTTCTGCTTTTCGAC	This work
*recA*2-Fw	GGCGAGGGTGTTTCCAAGAC	This work
*recA*2-Rv	GACGCTGGCTGTTATAGGAGAAC	This work
16SEc-F1	CCATGCCGCGTGTATGAAGAAG	This work
16SEc-R1	TCTGCGGGTAACGTCAATGAGC	This work
*pssA*G1f	CGCACATGCGAAAGATTTGCTGCG	This work
*pssA*2r	CCAGATCGAGGAATTCCCGACGTA	This work
*pssY*5f	GTCGTCGATGACGATGCGGCTGTT	This work
*pssY*5r	GAAACTATGTGCTTCCCATGTCATCG	This work

^1^ The sequences for the EcoRI, BamHI, XbaI, HindIII, and PstI restriction sites are underlined.

**Table 3:** Minimal free energy (ΔG) of secondary structures of the wild type *rosR* transcripts and their derivatives containing mutations in IR motifs.

Type of Transcripts	ΔG of Secondary Structures of *rosR* Transcripts (kcal/mol)	
	766 nt Long Transcript	733 nt Long Transcript
Wild type (control)	−348.89	−334.87
Mutation in the 5′-part of IR5	−347.84	−331.50
Mutation in the 3′-part of IR5	−334.05	−330.29
Mutation in the 5′-part of the RosR-box	−347.00	−333.25
Mutation in the 3′-part of the RosR-box	−348.92	−334.90
Mutation in the 5′-part of IR6	−345.92	−332.24
Mutation in the 3′-part of IR6	−346.72	−331.38
